# High prevalence of liver fibrosis among general population: a Romanian population-based study

**DOI:** 10.1097/HC9.0000000000000032

**Published:** 2023-01-18

**Authors:** Anca Trifan, Cristina-Maria Muzica, Robert Nastasa, Sebastian Zenovia, Ermina Stratina, Remus Stafie, Adrian Rotaru, Ana-Maria Singeap, Camelia Cojocariu, Catalin Sfarti, Irina Girleanu, Stefan Chiriac, Tudor Cuciureanu, Laura Huiban, Carol Stanciu

**Affiliations:** 1 Department of Gastroenterology, Grigore T. Popa University of Medicine and Pharmacy, Iasi, Romania; 2 “St. Spiridon” Emergency Hospital, Institute of Gastroenterology and Hepatology, Iasi, Romania

## Abstract

Although high mortality is associated with liver cirrhosis, patients usually have a good quality of life in the compensated phase, and the disease may progress undiagnosed for many years. Vibration-controlled transient elastography with controlled attenuation parameter is a useful noninvasive tool used to estimate both the severity of fibrosis and steatosis. Hence, we aimed to establish the prevalence of significant liver fibrosis diagnosed by vibration-controlled transient elastography in an apparently healthy population. Between December 2021 and March 2022, we conducted a prospective screening of liver fibrosis in apparently healthy participants from different counties of Northeastern Romania. All subjects’ medical history was recorded through a comprehensive questionnaire and underwent a liver stiffness measurement. Participants with abnormal liver stiffness measurement values were further evaluated by laboratory tests to identify the etiology of chronic liver disease. A total of 127 apparently healthy subjects were enrolled, mainly females (59.8%), with a mean age of 56±11 years. Overall, 12.6% of participants were found to have significant to advanced fibrosis, and 5.4% had liver cirrhosis. Among 184 participants with clinically significant fibrosis (≥8.0 kPa), 26.1% had a history of heavy alcohol intake, 22.3% tested positive for hepatitis B and C infection, and 2.1% with other etiologies. The remaining 49.5% participants with clinically significant fibrosis were diagnosed with NAFLD, with a mean controlled attenuation parameter value of 282±34 dB/m. The high prevalence of significant liver fibrosis in the general population of Romania is alarming and should raise awareness among clinicians and public health systems. Vibration-controlled transient elastography has demonstrated its usefulness as a screening tool to identify advanced liver fibrosis in general population and should be used in liver disease prevention strategies.

## INTRODUCTION

Liver cirrhosis is a major cause of morbidity and mortality worldwide and represents the leading cause of liver-related deaths, which entails a great burden for healthcare systems.^1^ Cirrhosis is the end-stage of liver progressive fibrosis, but due to its asymptomatic presentation in the initial stages, the prevalence of compensated cirrhosis is underestimated.^2^ Accordingly, a great number of patients with compensated cirrhosis remain undiagnosed until the first episode of decompensation occurs (ie, ascites, increased bilirubin levels, variceal bleeding, or encephalopathy). Even though this category of patients is usually promptly linked to medical care, the mortality and morbidity rates are far more raised than in compensated cirrhosis, with a 1-year case-fatality rate that can exceed 80% in some cases.^3^

The major causes of liver cirrhosis are currently represented by chronic HBV and HCV infections, NAFLD, and alcohol-related liver disease (ALD).^4^ Given the rapidly changing landscape of the liver cirrhosis etiology as a result of effective antiviral treatments and successful implementation of vaccination programs for viral hepatitis, NAFLD has become the most common cause of chronic liver disease worldwide, affecting ≥25% of the global adult population, with rising morbidity and mortality worldwide.^5^ In contrast, alcohol abuse still remains a major health problem, with ∼75 million individuals worldwide having an alcohol use disorder and a high risk of ALD.^6^ ALD is an umbrella term covering the alcoholic fatty liver, alcoholic hepatitis, and liver cirrhosis, but the majority of ALDs are frequently diagnosed at advanced stages, thus data on the prevalence rates and clinicopathological features of patients with early disease are scarce.

According to the latest epidemiological studies, it seems that the highest burden of liver disease in the world is in Europe, and worrying expectations regarding the increase in the number of cases in the coming years are being predicted.^7^ According to the latest World Health Organization (WHO) data published in 2018, the liver disease-related deaths in Romania reached 8763, or 3.75% of total deaths. The age-adjusted death rate is 26.90 per 100,000 population, which ranks Romania 47th in the world (https://www.worldlifeexpectancy.com/romania-liver-disease). Data from the Romanian National Institute for Public Health shows that in 2019 the leading cause of mortality in digestive diseases was liver cirrhosis, accounting for 34.6 deaths per 100,000 (https://insp.gov.ro/download/cnsisp/Fisiere-de-pe-site-CNSISP/buletine_informative_-_cauze_de_deces_in_romania/Buletin-Informativ-CAUZE-DECES-2018_2019.pdf). Similar mortality rates were seen in Lithuania and Hungary, with >20 deaths per 100,000.^7^

As liver cirrhosis ranks the 11th most frequent cause of death worldwide, and the seventh among the common causes of high disability-adjusted life years, there is an important negative financial impact on healthcare systems.^8^,^9^ The early detection of chronic liver diseases could improve the outcome of the patients and thus, reducing the burden of the disease by lowering the prevalence of liver cirrhosis and its complications, including liver failure, HCC, and death. To be reliable, this strategy requires the use of easy, accessible, and financially affordable noninvasive methods for the assessment of liver fibrosis in large populations of asymptomatic individuals. There are several serological markers that could predict future development of cirrhosis and advanced liver disease in the general population such as [aspartate aminotransferase (AST)–to-platelet ratio index, Fibrosis-4 Index (FIB-4), BARD, Forns, and NAFLD Score]. However, as shown by Hagstrom et al.^10^ in a recent study, their diagnostic performance is a modest one, with an area under the receiver operating characteristic curve between 0.54 and 0.71. Vibration-controlled transient elastography (VCTE) with controlled attenuation parameter (CAP) is a useful noninvasive tool used to estimate both the severity of fibrosis and steatosis, with high accuracy and acceptability worldwide. Moreover, has been applied as a screening tool in countries all over the world such as China, UK, France, Spain Italy, and The Netherlands involving >10,000 subjects from different population studies.^11^–^16^

Despite all data indicating an urgent need for the early detection of chronic liver disease, there are very few studies that performed a fibrosis screening in asymptomatic individuals, especially in Romania, where there is a very high prevalence of liver cirrhosis and, implicitly, an increased rate of liver-related deaths. Thus, we aimed to establish the prevalence of significant liver fibrosis diagnosed by VCTE in an apparently healthy population from Northeastern Romania.

## MATERIALS AND METHODS

### Study cohort

**FIGURE 1:**
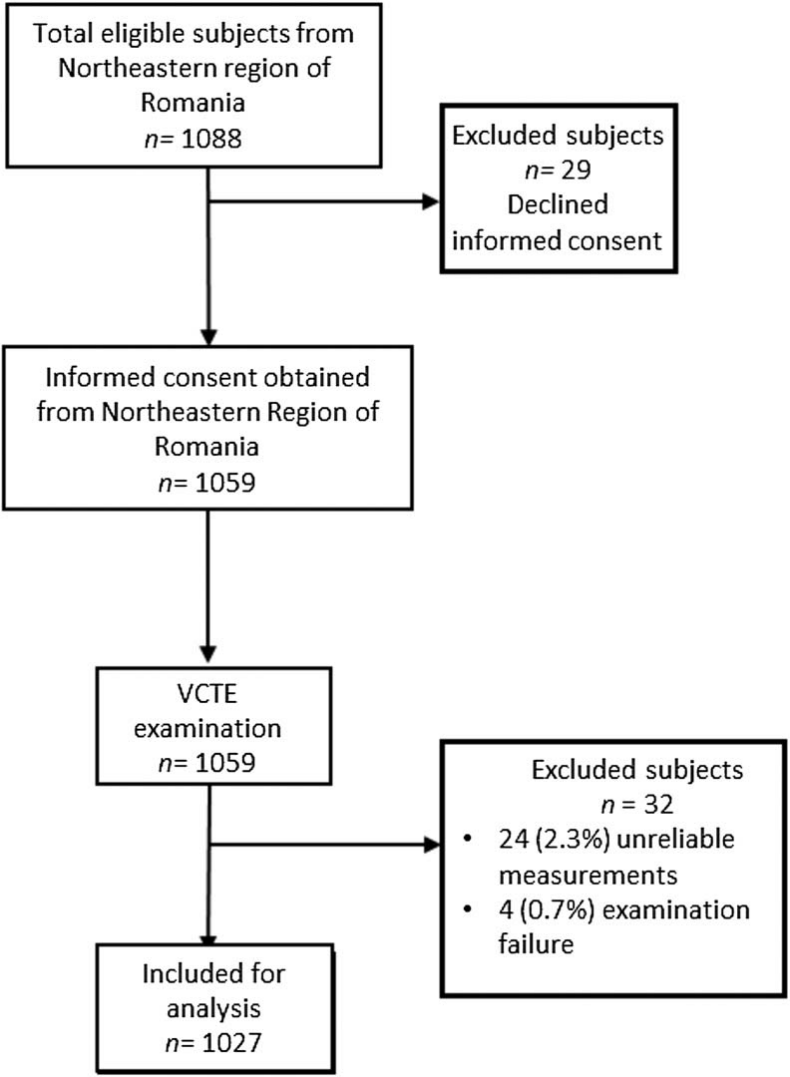
Study flowchart. A total of 61 patients from the Northeastern region of Romania were excluded from the study. Abbreviation: VCTE, vibration-controlled transient elastography.

From the total of 1088 eligible subjects, 1059 of them agreed to participate in the study and were screened using VCTE and CAP. A total of 32 patients were excluded: 24 (2.3%) had unreliable liver stiffness measurement (LSM) and 8 (0.8%) examination failure. This prospective study included 1027 apparently healthy participants from different areas of Northeastern Romania who were enrolled between December 2021 and March 2022 (Figure 1). The NorthEastern region of Romania is characterized by vulnerable conditions for several points, including greater exposure to risk factors like the use of tobacco products, consumption of energy-dense and high-fat food, heavy alcohol intake, physical inactivity, the high body mass index (BMI ≥25 kg/m^2^) of subjects, and decreased access to health services. All subjects enrolled in this study had more than 18 years old, had no past history of chronic liver disease and agreed to participate in the study by signing the informed consent. All patients that with a positive history of chronic HBV/HVC/HVD infection or other causes of chronic liver diseases (autoimmune hepatitis, Wilson disease, hemochromatosis, primary biliary cirrhosis, right-sided heart failure, HIV co-infection, alcoholic liver disease), existence of pregnancy, cardiac pacemakers, malignancy or end-stage renal diseases, and unreliable or failure VCTE measurements, were excluded. Moreover, from all the patients included in the study were recorded the height and weight measurements using a height meter and the weight scale. The cutoff values for lean (≥18.5 kg/m^2^), overweight (≥25 kg/m^2^), and obese (>30 kg/m^2^) subjects were defined by the WHO.^17^ The study activities were carried out in accordance with the principles of the Declarations of Helsinkis and Istanbul and was ratified by the Ethics Committee of our Institute and the University of Medicine and Pharmacy “Gr. T. Popa”, lasi, approval number 174. The informed consent was obtained from all participants before the examination.

### VTCE with CAP measurements and abdominal ultrasound

VCTE and CAP were performed using the FibroScan 502 Touch device (Echosens), equipped with M probe (standard probe—transducer frequency 3.5 MHz) and XL probe (transducer frequency 2.5 MHz) probe. All the examinations were carried out by an experienced physician, with >300 examinations in the past, following the procedure instruction.^18^ According to machine indications after at least 4 hours of fasting, participants were placed in a supine position with the right upper extremity at maximum abduction, and the LSM were taken on the right liver lobe for scanning through the intercostal according to guidelines recommendations.^19^ Basically, the examination started using the M probe, while the XL probe was automatically used in obese patients when the distance between skin-to-liver capsule was higher than 25 mm. Only individuals with minimum than 10 valid measurements and an interquartile range/median ratio, which does not exceed 30% (interquartile range/median≤30%) were included in the final analysis. LSM results were expressed in kilopascals (kPa) ranging from 1.5 to 75 kPa, and the liver fibrosis stages were distinguished by the following cutoff values: (mild fibrosis) ≥5.6 kPa, (significant fibrosis) ≥8.0 kPa, (advanced fibrosis) ≥9.6 kPa, and (cirrhosis) ≥13 kPa^12^; CAP was expressed in decibels/m, ranging from 100 to 400 dB/m, and the steatosis degree were differentiated by the following cutoffs: mild steatosis ≥274 dB/m, moderate steatosis ≥290 dB/m, severe steatosis ≥302 dB/m.^20^

Abdominal ultrasound scanning was performed after VCTE examinations by a single expert physician who is specialized in liver imaging blinded to all clinical data, with a 3.5–5 MHz convex probe and a high-resolution B-mode scanner (Supersonic Aixplorer MACH 30). The presence and severity of fatty liver was evaluated according to 4 ultrasonographic findings (hepatorenal contrast, bright liver, deep attenuation, and vessel blurring) using US fatty liver scoring system which classified the participants according to fatty liver grade in 3 groups: nonfatty liver group (0 points); mild fatty liver group (1–3 points); and fatty liver group (4–6 points).^21^

### Clinical and laboratory assessment

All the subjects underwent physical examination, anthropometric measurements laboratory tests, and FibroScan assessments in the same day. The following data concerning: demographics (sex, age), daily alcohol intake, medical history, comorbidities, type and duration of drug use, BMI, waist circumference, and systolic and diastolic blood pressure were established at the medical check-up visit.

All subjects with clinically significant fibrosis (≥8 kPa) were further evaluated by blood tests: hemoglobin, platelet count, international normalized ratio (INR), fibrinogen, C-reactive protein, ferritin, alanine aminotransaminase, AST, gamma-glutamyltransferase (GGT), alkaline phosphatase, total bilirubin, and the conjugated form, albumin, total cholesterol, triglycerides, LDL-cholesterol, HDL-cholesterol, HBsAg, and anti-HCVAb. Moreover, for every patient with clinically significant fibrosis, we calculated FIB-4 index, which had better performances than other simple noninvasive fibrosis tests in head-to-head comparisons, particularly in NAFLD subjects.^19^ In this study, NAFLD was defined as fatty liver diagnosed by ultrasonography or VCTE with CAP in a nondrinker subject (>30 g/d of alcohol for men and 20 g/d for women) who was not found with other etiology of chronic liver disease (chronic viral hepatitis, alcoholic liver disease, autoimmune hepatitis, Wilson disease, hemochromatosis). Also, all the subjects with an LSM cutoff value ≥8 kPa completed the AUDIT-C questionnaire to establish the alcohol consumption. The threshold that rules in subjects with excessive alcohol intake usually is 20 g per day in women, and 30 g per day in men according to recent scientific recommendations.^5^,^7^

### Statistical analysis

The quantitative variables were expressed as numbers, while the continuous variables were expressed as mean±SD. Distribution analysis was performed using the Kolmogorov-Smirnov test, while the parametric tests, such as *t* test and ANOVA, were used for the evaluation of differences between numerical variables with normal distribution. The Mann-Whitney test was used for variables with non-normal distribution such as the differences of the CAP values according to etiology of liver fibrosis. For identifying the risk factors associated with CAP and LSM values, we performed the univariate linear regression, which was followed by multivariate linear regression using only the significant factors. The 2-sided values of α < 0.05 (*p* < 0.05) were considered statistically significant. All the statistical parameters were achieved using IBM SPSS, Version 22.0 (IBM SPSS Inc.).

## RESULTS

### Characteristics of the study population

In the final analysis, we included 1027 patients who met the admission criteria. Of these patients with a mean age of 53.1±13.92 years, 754 (73.4%) of them were evaluated by the M probe, and 273 (26.6%) were evaluated by the XL probe. All baseline characteristics are presented in. Most of the participants had a BMI ≥25 kg/m^2^ (66.8%) with an increased percentage of females (53%). Hypertension and type 2 diabetes mellitus (T2DM) were present in 322 (31.4%) and 229 (22.3%) of patients, respectively. Of 1027 screened subjects, the median LSM value was 6.32±4.01 kPa, and according to VCTE measurements 500 (48.7%) of them had no fibrosis, 343 (33.4%) had mild fibrosis, 72 (7%) had significant fibrosis, 57 had advanced fibrosis (5.6%) and 55 (5.4%) had cirrhosis. Individuals with LSM value ≥8.0 kPa were predominantly females (59.8%), older (mean age: 56.46±12.72 y), and presented severe steatosis in a percentage of 63% versus 38.7% comparing to patients with a cutoff value <8.0 kPa (Table 1).

**TABLE 1:** General characteristics of study population

	Overall cohort (N = 1027) [n (%)]
Age (mean±SD) (y)	53.10±13.59
Females	544 (53.0)
Weight (mean±SD)	79.12±16.03
Height (mean±SD)	168.5±9.69
BMI (mean±SD) (kg/m^2^)	27.53±4.59
Underweight	20 (1.9)
Lean subjects	321 (31.3)
Overweight	394 (38.4)
Obese	292 (28.4)
Steatosis degree (dB/m)	
CAP < 274	553 (53.8)
CAP ≥ 274	89 (8.7)
CAP ≥ 290	87 (8.5)
CAP ≥ 302	298 (29.0)
Fibrosis stage (kPa)	
LSM < 5.6	500 (48.7)
LSM ≥ 5.6	343 (33.4)
LSM ≥ 8	72 (7.0)
LSM ≥ 9.6	57 (5.6)
LSM ≥ 13	55 (5.4)
CAP (mean±SD) (dB/m)	266.35±66.34
LSM (mean±SD) (kPa)	6.32±4.0
M probe	754 (73.4)

Abbreviations: BMI, body mass index; CAP, controlled attenuation parameter; LSM, liver stiffness measurement.

Also, the mean CAP score according to fibrosis stage, raised progressively from 243.25±62.11 dB/m in patients with LSM ≤5.5 kPa, 285.21±59.76 dB/m in patients with LSM <8.0 kPa, 290.15±64.07 dB/m in patients with LSM ≥8 kPa, 292.67±72.58 dB/m in patients with LSM ≥9.6 kPa, 300.24±68.03 dB/m among patients with cirrhosis (≥13 kPa). In addition, the proportion of overweight patients was higher (38.4% vs. 31.3% vs. 28.4%), in comparison with lean and obese subjects. Only a small proportion of patients (1.9%) had an BMI ≤17.5 kg/m^2^. Moreover, the presence of liver steatosis was found in 474 subjects (46.2%), of which 89 (18.8%) had mild steatosis, 87 (18.3%) had moderate steatosis, and 298 (62.9%) had severe steatosis, with a median CAP score of 266.35±66.34 dB/m among entire cohort (Table 1). Moreover, the mean LSM values according to steatosis degree was 5.47±3.31 kPa in group of patients without steatosis (S0), 6.66±5.53 kPa in mild steatosis, 6.62±3.45 kPa in moderate steatosis, and 7.70±4.38 kPa in severe steatosis.

### Biological parameters according to liver fibrosis stage

The subjects with a LSM value ≥8.0 kPa were referred to tertiary hepatology center for a consultation. According to biological parameters, patients with a cutoff value ≥8 kPa had low values of albumin (*p* = 0.002), platelet count (*p*=0.009), and HDL-C (*p*<0.001). Moreover, these individuals had increased values of AST (*p*<0.001), alanine aminotransaminase (*p*<0.001), GGT (*p*=0.043), alkaline phosphatase (*p*=0.047), C-reactive protein (*p*<0.001), and Moreover, 91 (49.5%) had NAFLD, 48 (26.1) had alcohol liver disease, 25 (13.6%) of the subjects were identified with HVB, 14 (7.6%) with HVC, and 6 (3.2%) with other etiologies (2 with HVB+HVD, 2 with AIH, 1 with Hemochromatosis, and 1 with Wilson disease). Among cirrhotic group, the presence of NAFLD was found in 19 (34.5%) individuals, 18 (32.7%) had ALD, 17 (30.9%) had viral hepatitis and 1 (1.8%) had Wilson disease. Based on CAP measurements, most of the patients with a LSM ≥13 kPa had severe steatosis compared with those with advanced and significant fibrosis (70.9% vs.61.4% vs. 58.3%), respectively. Moreover, mean CAP value increased from 293.6±62.53 dB/m in subjects with significant fibrosis to 305.24±62.19 dB/m in cirrhotic group. In addition, the mean value of FIB-4 index was 1.80±1.13 without any statistical differences among groups (*p*=0.061) (Supplemental Table 2, http://links.lww.com/HC9/A87).

### Characteristics of patients with ≥significant liver fibrosis

Clinical and biological parameters of patients with ≥significant liver fibrosis were summarized in Table 2. Most of the patients with a LSM ≥8 kPa were females, and the proportion increased according to fibrosis stage (*p*=0.024). Regarding biological parameters, subjects with cirrhosis had higher values of alanine aminotransaminase (*p*<0.001), AST (*p*<0.001), alkaline phosphatase (*p*<0.001), GGT (*p*<0.001), and FIB-4 index (*p*<0.001). Also, they had and decreased values of platelet count (*p*<0.001), and INR (*p*<0.001). The mean LSM value increased rapidly from 8.55±0.48 kPa among patients with significant fibrosis to 19.39±7.11 kPa in the cirrhotic group (*p*<0.001). Concerning the mean CAP score, there was a slightly increased from 293.6±62.53 dB/m among patients with a LSM ≥13 kPa, to 305.24±62.65 in subjects with cirrhosis, but with no statistical difference (*p*=0.0742). Also, the percentage of patients with a high FIB-4 index (≥1.30) was increased from 54.2% in those with significant liver fibrosis (≥8 kPa) to 72.7% in the cirrhotic group (≥13 kPa) (*p*=0.026) (Table 2). We included the analysis of LSM and FIB-4 cutoff values in a flowchart. Approximately, half of the patients (45.7%) with a low risk for liver fibrosis (FIB-4<1.3), had clinically significant fibrosis (≥8 kPa), [33 (39.3%) with a LSM ≥8 kPa, 36 (42.9%) with a LSM (≥9.6 kPa), 15 (17.8%) with a LSM (≥13 kPa)] would have been excluded from VCTE examination if we performed only FIB-4 index test according to liver fibrosis cutoff values of FIB-4 index. Moreover, only 39.3% of the patients with a low risk of liver fibrosis according to the FIB-4 index (FIB-4<1.3) could have skipped VCTE examinations if we used the 2-tier approach (Supplemental Figure 2, http://links.lww.com/HC9/A87).

**TABLE 2:** Baseline characteristics of patients according to fibrosis stage

	Mean±SD or n (%)			
	LSM ≥8 kPa [72 (39.1%)]	LSM ≥9.6 kPa [57 (31%)]	LSM ≥13 kPa [55 (29.9%)]	*p*
Sex (female)	37 (51.4)	32 (56.1)	41 (74.5)	0.024
Age (y)	54.78±12.97	55.46±13.4	59.71±11.18	0.074
BMI (kg/m^2^)	28.16±5.93	29.12±4.29	27.68±5.64	0.357
T2DM	14 (19.4)	19 (33.3)	13 (23.6)	0.190
Underweight	3 (4.2)	0 (0)	0 (0)	0.124
Normal weight	18 (25)	10 (17.5)	19 (34.5)	
Overweight	31 (43.1)	28 (49.1)	24 (43.6)	
Obesity	20 (27.8)	19 (33.3)	12 (21.8)	
Hypertension	22 (30.6)	17 (29.8)	18 (32.7)	0.943
Platelet count (G/L)	220.43±69.1	234.68±59.37	192.3±62.65	<0.001
INR	1.10±0.11	1.01±0.12	1.12±0.2	<0.001
CRP (mg/dL)	0.54±0.35	0.51±0.4	0.63±0.57	0.301
Ferritin (mg/dL)	165.25±92.95	144.82±75.66	176.52±88.79	0.147
ALT (IU/L)	41.61±28.05	43.7±27.62	62.67±34.7	<0.001
AST (IU/L)	37.62±20.53	34.49±20.19	51.9±24.51	<0.001
GGT (IU/L)	64.30±56.28	43.19±23.96	81.52±66.21	0.001
ALP (IU/L)	103.28±42.78	79.75±30.14	107.27±45.25	<0.001
Bilirubin (mg/dL)	2.08±1.08	0.72±0.26	0.83±0.44	0.455
Albumin (g/dL)	5.66±0.94	4.61±0.21	4.32±0.45	0.401
Fasting glucose (mg/dL)	110.05±32.87	126.4±47.88	119.16±38.99	0.068
Cholesterol (mg/dL)	224.08±54.77	218.89±34.11	230.58±49.58	0.431
Triglycerides (mg/dL)	153.73±47.94	157.19±61.46	168.56±56.86	0.308
LDL-cholesterol (mg/dL)	133.45±43.81	147.03±35.31	138.78±40.65	0.168
HDL-cholesterol (mg/dL)	44.38±12.74	40.24±10.41	43.72±10.79	0.107
CAP (dB/m)	293.6±62.53	292±72.58	305.24±62.65	0.072
Steatosis degree				0.458
CAP < 274 dB/m	20 (27.8)	14 (24.6)	12 (21.8)	
CAP ≥ 274 dB/m	6 (8.3)	1 (7)	2 (3.6)	
CAP ≥ 290 dB/m	4 (5.6)	4 (7)	2 (3.6)	
CAP ≥ 302 dB/m	42 (58.3)	35 (61.4)	39 (70.9)	
LSM (kPa)	8.55±0.48	10.6±0.86	19.39±7.11	<0.001
FIB-4 index	1.27±0.67	1.74±1.19	2.44±1.63	<0.001
FIB-4 index >1.3	39 (54.2)	36 (63.6)	40 (72.7)	0.026

Abbreviations: ALP, alkaline phosphatase; ALT, alanine aminotransferase; AST, aspartate aminotransferase; BMI, body mass index; CAP, controlled attenuation parameter; CRP, C-reactive protein; FIB-4, Fibrosis-4 Index; GGT, gamma-glutamyl transferase; INR, international normalized ratio; LSM, liver stiffness measurement; T2DM, type 2 diabetes mellitus.

### Factors associated with increased LSM and CAP values

We carried out a univariate linear regression analysis to notice risk factors associated with increased LSM and CAP values, and only those with a significant *p* value (*p*<0.05) were included in the multivariate regression analysis (Table 3). In the univariate analysis, we observed that age (β=0.123, *p*=0.004), INR (β=0.167, *p*=0.023), platelets (β=−0.307, *p*<0.001), alkaline phosphatase (β=0.297, *p*<0.001), GGT (β=0.350, *p*<0.001), alanine aminotransferase (β=0.149, *p*=0.044), AST (β=0.306, *p*<0.001), FIB-4 index (β=0.363, *p*<0.001), and presence of T2DM (β=0.166, *p*=0.017) were risk factors associated with LSM value in all patients. From these factors, in the multivariate analysis, only age (β=0.137, *p*=0.025), platelets (β=−0.257, *p*=0.032), and GGT (β=0.284, *p*<0.001) were independently associated with increased LSM value. Regarding, increased CAP values we identify that cholesterol (β=−0.159, *p*=0.031), BMI (β=0.393, *p*<0.001), INR (β=−0.148, *p*=0.045), C-reactive protein (β=0.164, *p*=0.026), ferritin (β=0.223, *p*=0.002) and fasting glucose (β=0.255, *p*<0.001), and presence of T2DM (β=0.281, *p*<0.001) were risk factors associated independently in univariate analyses. Moreover, BMI (β=0.411, *p*<0.001), INR (β=−0.251, *p*=0.008), C-reactive protein (β=0.105, *p*=0.023), ferritin (β=0.157, *p*=0.012), fasting glucose (β=0.375, *p*=0.002), and T2DM (β=0.322, *p*<0.001) were associated independently with increased CAP score also in multivariate linear regression (Table 3).

**TABLE 3:** Univariate and multivariate linear regression analyses of factors associated with increased LSM and CAP values

	LSM (kPa)				CAP (dB/m)			
	Univariate		Multivariate		Univariate		Multivariate	
Parameters	β	*p*	β	*p*	β	*p*	β	*p*
Age (y)	0.123	0.004	0.137	0.025	0.045	0.540		
BMI (kg/m^2^)	−0.096	0.193			0.393	<0.001	0.411	<0.001
T2DM	0.166	0.017	0.141	0.161	0.281	<0.001	0.322	<0.001
CAP (dB/m)	0.034	0.643			—	—	—	—
Platelets (G/L)	−0.307	<0.001	−0.257	0.032	−0.003	0.963		
INR	0.167	0.023	0.129	0.433	−0.148	0.045	−0.251	0.008
CRP (mg/dL)	0.097	0.192			0.164	0.026	0.105	0.023
Ferritin (mg/dL)	0.069	0.355			0.223	0.002	0.157	0.012
Fasting glucose (mg/dL)	0.121	0.102			0.255	0.001	0.375	0.002
ALT (IU/L)	0.297	<0.001	0.101	0.377	0.086	0.243		
AST (IU/L)	0.306	<0.001	0.206	0.073	0.061	0.409		
GGT (IU/L)	0.350	<0.001	0.284	<0.001	0.046	0.535		
ALP (IU/L)	0.149	0.044	−0.109	0.503	0.068	0.359		
Bilirubin (mg/dL)	−0.040	0.589			0.120	0.106		
Cholesterol (mg/dL)	0.047	0.525			−0.159	0.031	−0.252	0.036
Triglycerides (mg/dL)	0.077	0.301			−0.126	0.088		
Albumin (g/dL)	−0.061	0.414			0.118	0.112		
LDL-cholesterol (mg/dL)	−0.047	0.529			−0.011	0.881		
HDL-cholesterol (mg/dL)	−0.084	0.259			0.120	0.106		
FIB-4 index	0. 363	<0.001	0.115	0.088	0.115	0.206		

Abbreviations: ALP, alkaline phosphatase; ALT, alanine aminotransferase; AST, aspartate aminotransferase; BMI, body mass index; CAP, controlled attenuation parameter; CRP, C-reactive protein; FIB-4, Fibrosis-4 Index; GGT, gamma-glutamyl transferase; INR, international normalized ratio; LSM, liver stiffness measurement; T2DM, type 2 diabetes mellitus.

### Prevalence of liver fibrosis and steatosis according to age group

The prevalence rate of liver fibrosis and steatosis for each age group was summarized in Table 4. The overall prevalence of ≥significant liver fibrosis increased rapidly with age in the population study from 10.4% in the group of 18–40 years old, to 17.8% in the group of 41–60 years old, and 21% in the age group of 61–74 years old, reaching 28.9% among those aged ≥75 years. We also found a significant correlation between age and increased LSM (*r*=0.045, *p*<0.001). Regarding liver steatosis, the prevalence rates were more common found among patients aged between 61 and 74 years old (71.3%) and those aged between 41 and 60 years old (68.3%). Subjects aged ≥75 years had a prevalence of liver steatosis of 61.6% and the young group aged between 18 and 40 years old had the lowest prevalence hepatic steatosis (36.3%). Moreover, the presence of overweight and obesity was more often found in patients aged between 61 and 74 years old, with a prevalence of 38.5% and 36.8%, respectively (Table 4).

**TABLE 4:** Baseline characteristics of patients according to age group

	n (%)				
Parameters	18–40 y (n=182)	41–60 y (n=497)	61–74 y (n=296)	≥75 y (n=52)	*p*
Male sex	176 (100)	297 (59.8)	4 (1.4)	52 (100)	<0.001
BMI (mean±SD) (kg/m^2^)	25.68±4.57	27.64±4.52	28.51±4.49	27.48±4.28	<0.001
Underweight	6 (3.4)	10 (2)	4 (1.4)	0 (0)	<0.001
Lean	88 (50)	143 (28.8)	69 (23.3)	18 (34.6)	
Overweight	51 (29)	203 (40.8)	114 (38.5)	23 (44.2)	
Obese	31 (17.6)	141 (28.4)	109 (36.8)	11 (21.2)	
LSM (mean±SD) (kPa)	5.39±2.65	6.14±3.09	6.97±5.45	7.40±5.08	<0.001
CAP (mean±SD) (dB/m)	228.65±68.46	273.45±64.15	278.33±60.17	265.65±68.26	<0.001
Steatosis degree					<0.001
CAP <274 dB/m	145 (79.7)	249 (50.1)	131 (44.3)	28 (53.8)	
CAP ≥274 dB/m	8 (4.4)	43 (8.7)	35 (11.8)	3 (5.8)	
CAP ≥290 dB/m	8 (4.4)	41 (8.2)	34 (11.5)	4 (7.7)	
CAP ≥302 dB/m	21 (11.5)	164 (33)	96 (32.4)	17 (32.7)	
Fibrosis stages					<0.001
LSM <5.6 kPa	111 (61)	230 (46.3)	138 (46.6)	21 (40.4)	
LSM ≥5.6 kPa	52 (28.6)	179 (36)	96 (32.4)	16 (30.8)	
LSM ≥8 kPa	10 (5.5)	36 (7.2)	23 (7.8)	3 (5.8)	
LSM ≥9.6 kPa	5 (2.7)	32 (6.4)	13 (4.4)	7 (13.5)	
LSM ≥13 kPa	4 (2.2)	20 (4)	26 (8.8)	5 (9.6)	

Abbreviations: BMI, body mass index; CAP, controlled attenuation parameter; LSM, liver stiffness measurement.

### LSM and CAP values according to etiology of chronic liver disease

**FIGURE 2:**
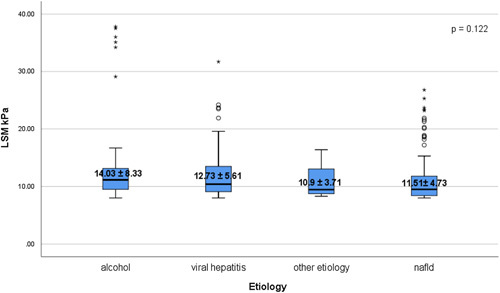
Distribution of LSM values according to etiology. The bottom and the top of each box represent the 25th and 75th percentiles, while the lines through the box indicate the median. Abbreviation: LSM, liver stiffness measurement.

**FIGURE 3:**
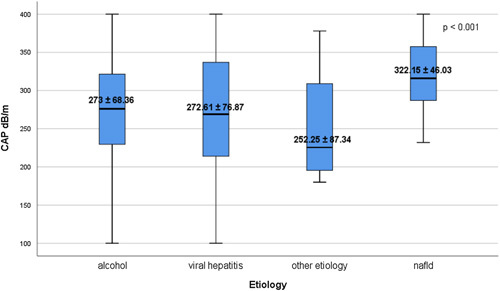
Distribution of CAP values according to etiology. The bottom and top of each box represent the 25th and 75th percentiles, while the lines through the box indicate the median. Abbreviation: CAP, controlled attenuation parameter.

According to each etiology, the mean LSM values ranged from 10.9±3.71 kPa among group of patients with other etiology, to 11.51±4.73 kPa in subjects with NAFLD, 12.73±5.61 kPa among individuals with viral hepatitis and 14.03±8.33 kPa in subjects with alcohol liver disease, respectively, with an insignificant statistical differences of LSM values (*p*=0.122) (Figure 2). Furthermore, the mean CAP score increased rapidly from 252.25±87.34 dB/m in group of individuals with other etiology, to 272.61±76.87 dB/m in viral hepatitis group, 273±68.36 dB/m in subjects with alcohol liver disease, and 322.15±46.03 dB/m among NAFLD patients, with an important statistical difference of CAP score among these groups (*p*<0.001) (Figure 3).

## DISCUSSION

Considering the fact that liver cirrhosis is a major health problem globally, due to its high mortality and heavy financial burden, there is an urgent demand to change the pattern of diagnosis of chronic liver disease from late stages (ie, complications of cirrhosis), to early diagnosis (ie, advanced fibrosis or compensated cirrhosis).^22^ This new attitude would call for identification of asymptomatic individuals using noninvasive tools for evaluation of liver fibrosis in general population like VCTE.^4^

An important aspect of the screening procedure is the economic efficiency, and an important lesson learned from cancer screening is the selection of individuals with a high pretest probability. A recent study published by Serra-Burriel et al.^23^ found that the cost of using VCTE ranging from $6000 per quality-adjusted life-year in low-prevalence general population settings to $2000 per quality-adjusted life-year in at-risk population such as individuals with metabolic syndrome or heavy alcohol consumers. This costs being below the limit for making part to the portfolio of covered services in most developed regions ($100,000 in the US and between $25,000 and 50,000 in Europe).^24^–^28^ However, there is a demand for future initiatives for comparing existing noninvasive tests of fibrosis in terms of accuracy and applicability in specific settings, and evaluating the cost-effectiveness of screening, such as Renown (Nevada, USA), Scarred Liver Project (Nottingham, UK), SEAL (Germany), that are ongoing or finalized like LiverScreen (Europe).^4^,^29^

This study found that the prevalence of advanced fibrosis and cirrhosis was high in the general population using VCTE (5.6% and 5.4%, respectively) and approximately two third of them were presented with severe steatosis (66.1%). The following strengths of our study were: (1) the admission of large number of asymptomatic individuals (1027 subjects), (2) the use of VCTE for distinguishing of different stages of liver fibrosis, which is a reliable noninvasive method recommended by guidelines in health check-ups,^19^ (3) this study was the first epidemiological study of a randomly unselected patients from general population and provided valuable data in Romania.

Until now, there are very few reports regarding the prevalence of clinically significant (≥8 kPa) liver fibrosis in general population ranging from 2% to 13.8%.^11^–^13^,^16^,^30^–^33^ However, a precise cutoff value for dichotomization between different stages of liver fibrosis is not yet been defined. Chávez-Tapia and colleagues conducted a rural population study using TE as a screening method for liver fibrosis, founding a prevalence of 8.02% (7–9 kPa) for subjects with intermediate risk of cirrhosis, and 7.35% (>9 kPa) for high risk of cirrhosis participants. Although the presence of alcohol intake, T2DM, and obesity war correlated with an increased risk of cirrhosis.^34^

Caballeria and colleagues in a cross-sectional populational-based study noted a prevalence of 5.8%–3.6% raging with cutoff values from 8 to 9 kPa, respectively for significant liver fibrosis. Moreover, the authors highlighted that the best cutoff value for liver fibrosis was 9.2 kPa with a sensitivity of 93% and specificity of 78% for predicting significant fibrosis.^13^ Following the same idea, Serra-Burriel et al.^23^ found that a cutoff value of 9.1 kPa for liver fibrosis had the best accuracy for diagnosis of significant liver fibrosis in the general population in a cost-effectiveness analysis study, noting that transient elastography is a cost-effective tool for identifying subjects with liver fibrosis in primary care units. In our study, the prevalence of significant advanced liver fibrosis (≥8.0–12.9) was 12.6%, and for cirrhosis (≥13 kPa) was 5.3%, respectively. Moreover, predictive factors associated with increased liver fibrosis were females, hepatocytolisis syndrome, and CAP score. Also in the multivariate analysis, we found that increased LSM values are associated with older age, high and GGT levels. A strength of the current investigation compared with other studies is the homogeneity of the studied population regarding the etiologies of liver disease. On the contrary, Wong et al.^12^ conducted a study in an Asian population and reported a prevalence of advanced liver fibrosis of 2% using a similarly cutoff value for advanced fibrosis of (9.6 kPa) to our study. This difference could be explained by the fact that the population involved in the previous study excluded patients with chronic viral hepatitis, significant alcohol consumption, or other known liver diseases.

Among 1027 subjects included, the prevalence of steatosis using a cutoff for mild steatosis of ≥274 dB/m was 46.2%, from which 91 patients had clinically significant liver fibrosis (≥8 kPa), revealing a prevalence of 7.6% in this group. Our results are following those found by Petta et al.^15^ in a Mediterranean cohort study, in which the prevalence of NAFLD with advanced fibrosis was 6.5%. On the contrary, the results reported by Ciardullo et al.^35^ in a cross-sectional study, which involved 1710 participants from US general population, found a slightly decreased prevalence of NAFLD of 37.1% using a similar cutoff value for CAP score of ≥274 dB/m for mild steatosis. Other studies that included young adults with NAFLD, found the prevalence of clinically significant liver fibrosis at approximately 3%, suggesting that older age and high mean BMI are risk factors associated with increased LSM values.^36^,^37^ Our findings highlighted also the prevalence of NAFLD among patients with significant liver fibrosis (≥8 kPa) up to 49.5%, of which 20.9% are in the cirrhosis stage. This data cannot approximate the real prevalence of cirrhosis among NAFLD subjects from our studied population, but it emphasizes the main feature of this disease among patients with significant liver fibrosis.

The current study included patients with HBsAg and anti-HCVAb positive, which revealed that chronic viral hepatitis were a strong predictor of clinically significant fibrosis (≥8 kPa). Among these subjects, the prevalence of advanced fibrosis and cirrhosis was 57.2% in patients with anti-HCVAb positive and 64% HBsAg, respectively, with a mean LSM value of 12.73±5.61 kPa among these 2 etiologies. This findings suggest that viral hepatitis were still important risk factors for liver fibrosis in asymptomatic population, more often associated in individuals that 50s or 60s years old.^38^ In our research, we also included subjects with alcohol consumption observing that the prevalence of advanced fibrosis and cirrhosis was 75%, with a mean LSM value of 14.03±8.33 kPa. Moreover, hepatic steatosis occurs in most of the heavy drinkers (77.1%), with a mean CAP value of 273±68.36 dB/m. However, it must be mentioned that in many individuals there is an intertwined relationship between alcohol consumption and metabolic disorders.^39^–^41^ These findings suggest the need for early screening of liver fibrosis in subjects with excessive alcohol intake, perhaps at the time of the alcohol liver disease diagnosis to prevent further complications of cirrhosis.

The current research found that VCTE is a valuable method for distinguishing stages of liver fibrosis in general population (without known with chronic liver disease), having a higher efficacy for the screening of liver fibrosis when compared with other noninvasive tests. VCTE had a higher accuracy for detecting patients with significant (≥8 kPa), advanced liver fibrosis (≥9.6 kPa), and cirrhosis (≥13 kPa) compared with FIB-4 index which is has been extensively validated with easy accessibility.^19^ These differences can be explained by the fact that the performance of FIB-4 index to identify significant fibrosis is limited and the test should be used in patients with risk factors of advanced liver fibrosis (those with metabolic risk factors and/or excessive alcohol intake).^13^ There are several studies regarding the cost-effectiveness analyses of adopting the 2-tier approach (use of FIB-4 index followed by VCTE) which showed that testing populations at risk for liver disease but with low prevalence of advanced fibrosis is cost-beneficial.^19^,^42^–^44^ Crossan et al.^43^ in 2019, found that the sequential use of noninvasive tests in primary care is an effective way to reduce hepatological consultations and is associated with significant cost savings up to 40%. Another study published in 2020 by Nourredin et al.^44^ showed that the screening for NAFLD with a 2 steps approach in patients with T2DM is more cost-effective than not screening this population and must be started at a younger age for the increases of cost-beneficial. More than half (55.4%) of our patients had an lower risk of advanced fibrosis (LSM≥8 kPa) and cirrhosis (LSM ≥13 kPa) according to cutoff values of FIB-4 index of each age group,^45^ and approximately two third of patients (60.7%) with a value of FIB-4 index<1.3 had higher risk for liver fibrosis using VCTE with an LSM≥9.6 kPa. Also, 40 (58.8%) of patients with intermediate risk for liver fibrosis according to FIB-4 index (FIB-4≥1.3) had advanced fibrosis (LSM≥9.6 kPa) and cirrhosis (LSM≥13 kPa) using VCTE examinations. Instead, approximately one third (34.4%) of the patients with a high risk for liver fibrosis (FIB-4≥2.67) had only significant liver fibrosis (LSM≥8 kPa) according to VCTE. Our results could only speculate that the detection of liver fibrosis with VCTE in the general population with vulnerable conditions is a highly cost-effective strategy and potentially cost-saving in the era of NAFLD epidemics, because the 2-tier approach could miss patients with advanced fibrosis or cirrhosis. Although, the cost-efficiency analysis regarding this 2-tier approach compared with VCTE alone must be done in lower-middle income countries like Romania as well, with a necessity for further studies in this area.

Our study had some limitations. First of all, no histological information on individuals with clinically significant liver fibrosis was analyzed, due to the absence of performing liver biopsy because we enrolled apparently healthy subjects in our study. Moreover, another limitation of our research could be considered to be the fact that patients with clinically significant liver fibrosis did not undergo a second examination using different methods for liver fibrosis and steatosis assessment such as magnetic resonance elastography and 2-dimensional shear wave elastography. However, VCTE has been validated and recommended by guidelines over the years for fibrosis evaluation in chronic viral hepatitis as well as in NAFLD subjects. Third, there is no precise cutoff value for CAP score and liver stiffness globally, due to few studies conducted up to now. Fourth, there was no follow-up to determine the changes in liver stiffness or CAP score after applying interventions strategies and to monitor long-term outcomes.

## CONCLUSIONS

The prevalence of clinically significant liver fibrosis ≥8 kPa was increased among asymptomatic healthy individuals from the general population. Those with risk factors such as older age, male sex, obesity, T2DM, chronic viral hepatitis, and excessive alcohol intake had the highest prevalence. Moreover, most of the cases with clinically significant liver fibrosis were NAFLD subjects, highlighting the fact that NAFLD was the most common etiology of chronic liver disease. To prevent disease progression and decrease liver-related morbidity and mortality, screening for liver fibrosis should be considered using VCTE in the asymptomatic general population, particularly among those living in vulnerable conditions.

## Supplementary Material
