# Supplementary material for: High prevalence of liver fibrosis among general population: a Romanian population-based study
Source: Hepatol Commun. 2023 Jan 18;7(2):e0032. doi: 10.1097/HC9.0000000000000032 (PMC9851682; doi:10.1097/HC9.0000000000000032)
Supplement: Supplementary file 1 [file hc9-7-e0032-s001.docx]

|  | | | |  |  | | |  |  | | | |  | |  | |  |  |
| --- | --- | --- | --- | --- | --- | --- | --- | --- | --- | --- | --- | --- | --- | --- | --- | --- | --- | --- |
|  | **Participants ≥F2**  **n, 184** | | **Alcohol**  **n, 48 (26.1)** | | | | **NAFLD**  **n, 91 (49.5)** | | **HCV**  **n, 14 (7.6)** | **HBV**  **n, 25 (13.6)** | | | | **Other etiologies, n 6 (3.2)** | **p - value** | | |  |
| Gender (female), n (%) | 110 (59.8) | 17 (35.4) | | | | 60 (65.9) | | | 4 (28.6) | | 16 (64) | 3 (50) | | | **0.049** | | | |
| Age, yr. | 56.46 ± 12.72 | 55.69 ± 13.32 | | | | 57.92 ± 12.44 | | | 50.07±12.27 | 57.92±12.28 | | 49.33 ± 14.5 | | | 0.058 |  |  |  |
| BMI (kg/m^2^)  T2DM, n(%) | 28.32 ± 5.39  46 (25) | 27.06 ± 4.99  8 (16.7) | | | | 29.69 ± 5.02  22 (24.2) | | | 26.4±4.37  1 (7.1) | 27.35±7.08  17 (68) | | 26 ± 3.58  1 (16.7) | | | 0.053  0.608 |  |  |  |
| Underweight, n(%) | 3 (1.6) | 1 (2.1) | | | | 1 (1.1) | | | 1 (7.1) | 0 | | 0 (0) | | | 0.007 |  |  |  |
| Normal weight, n(%) | 47 (25.5) | 17 (35.4) | | | | 14 (15.4) | | | 4 (28.6) | 9 (36) | | 3 (50) | | |  |  |  |  |
| Overweight, n(%) | 83 (45.1) | 20 (41.7) | | | | 40 (44) | | | 8 (57.1) | 12 (48 | | 3 (50) | | |  |  |  |  |
| Obesity, n(%) | 51 (27.7) | 10 (20.8) | | | | 36 (39.6) | | | 1 (7.1) | 4 (16) | | 0 (0) | | |  |  |  |  |
| Hypertension, n(%) | 57 (31) | 9 (18.8) | | | | 27 (29.7) | | | 5 (35.7) | 11 (44) | | 2 (33.3) | | | 0.446 |  |  |  |
| Platelet count (G/L) | 219.53 ± 67.15 | 230.4 ± 75.12 | | | | 212.83 ± 68.18 | | | 230±67.89 | 218.04±44.02 | | 216.5 ± 69.07 | | | 0.891 |  |  |  |
| INR | 1.08 ± 0.15 | 1.05 ± 0.16 | | | | 1.08 ± 0.14 | | | 1.06±0.11 | 1.14±0.16 | | 1.11 ± 0.2 | | | 0.118 |  |  |  |
| CRP (mg/dl) | 0.56 ± 0.44 | 0.42 ± 0.31 | | | | 0.65 ± 0.43 | | | 0.52±0.4 | 0.55±0.63 | | 0.41 ± 0.35 | | | 0.108 |  |  |  |
| Ferritin (mg/dl) | 162.29 ± 87.12 | 154.43 ± 73.9 | | | | 171.24 ±88.87 | | | 174.4±101.89 | 156.96±97.51 | | 83.33 ± 38.1 | | | 0.373 |  |  |  |
| ALT (IU/L) | 48.55 ± 31.31 | 52.25 ± 31.95 | | | | 46.65 ± 32.65 | | | 51.85±24.01 | 47.96±31.28 | | 42.5 ± 24.87 | | | 0.788 |  |  |  |
| AST (IU/L) | 40.92 ± 22.78 | 43.56 ± 25.27 | | | | 40.82 ± 22.37 | | | 42.14±21.47 | 37.08±22.23 | | 34.5 ± 14.86 | | | 0.870 |  |  |  |
| GGT (IU/L) | 62.91 ± 54.07 | 81.27 ± 54.29 | | | | 50.49 ± 51.85 | | | 74.5±50.77 | 72.72±67.73 | | 44.83 ± 28.63 | | | 0.894 |  |  |  |
| ALP (IU/L) | 97.15 ± 41.61 | 95.77 ± 38.79 | | | | 99.11 ± 42.20 | | | 89.28±33.67 | 96.72±50.97 | | 99 ± 38.62 | | | 0.968 |  |  |  |
| Bilirubin (mg/dl) | 1.12 ± 0.68 | 1.2 ± 0.622 | | | | 0.79 ± 0.48 | | | 0.72±0.26 | 0.77±0.32 | | 0.99 ± 0.64 | | | 0.995 |  |  |  |
| Albumin (g/dl) | 4.94 ± 0.88 | 4.55 ± 0.42 | | | | 5.36 ± 0.86 | | | 4.57±0.22 | 4.47±0.47 | | 4.43 ± 0.33 | | | 0.996 |  |  |  |
| Fasting glucose (mg/dl) | 117.84 ± 40.21 | 109.37 ± 18.8 | | | | 117.41 ± 38.81 | | | 122.28±49.69 | 114.24±28.2 | | 118.5 ± 42.57 | | | 0.828 |  |  |  |
| Cholesterol (mg/dl) | 224.42 ± 47.61 | 213.7 ± 41.11 | | | | 248.03 ± 46.89 | | | 211.85±62.92 | 242.28±50.62 | | 201.66 ± 25.75 | | | 0.167 |  |  |  |
| Triglycerides (mg/dl) | 159.23 ± 55.14 | 164.81 ± 53.1 | | | | 177.16 ± 56.15 | | | 157.28±45.83 | 159±54.02 | | 178.33 ± 27.58 | | | 0.051 |  |  |  |
| LDL-C (mg/dl) | 139.25 ± 40.59 | 140.1 ± 33.8 | | | | 140.91 ± 41.98 | | | 121.92±39.77 | 142.76±51.09 | | 132.83 ± 13.65 | | | 0.856 |  |  |  |
| HDL-C (mg/dl) | 42.9 ± 11.57 | 44.06 ± 9.15 | | | | 39.62 ± 13.16 | | | 42.57±8.4 | 43.68±11.52 | | 42.5 ± 10.98 | | | 0.552 |  |  |  |
| CAP dB/m  Steatosis degree  CAP < 274dB/m, n(%) CAP ≥ 274 dB/m, n(%)  CAP ≥ 290 dB/m, n (%)  CAP ≥ 302 dB/m, n (%) | 296.79 ± 65.58  64 (34.8)  15 (8.2)  17 (9.2)  88 (47.8) | 225±48.28  24 (50)  4 (8.3)  5 (10.4)  15 (31.3) | | | | 322.15±46.03  11 (12.1)  8 (8.8)  9 (9.9)  63 (69.2) | | | 271.14±84.88  8 (57.1)  0 (0)  0 (0)  6 (42.9) | 272.12±74.71  12 (48)  1 (4)  2 (8)  10 (40) | | 264.5 ± 81.08  4 (66.7)  0 (0)  0 (0)  2 (33.3) | | | **<0.001**  **<0.001** |  |  |  |
| FIB – 4 index | 1.80 ± 1.3 | 1.68 ± 1.14 | | | | 1.98 ± 1.47 | | | 1.53 ± 1.08 | 1.62 ± 0.96 | | 1.55 ± 1.44 | | | 0.061 |  |  |  |
| LSM kPa | 12.43 ± 6.06 | 14.03 ± 8.33 | | | | 11.51 ± 4.73 | | | 12.97±5.35 | 12.3±5.21 | | 12.75 ± 9.63 | | | 0.353 |  |  |  |
| Fibrosis stage |  |  | | | |  | | |  |  | |  | | | 0.107 |  |  |  |
| LSM ≥ 8 kPa, n (%) | 72 (39.1) | 12 (25) | | | | 42 (46.2) | | | 6 (42.9) | 9 (36) | | 1 (16.7) | | |  |  |  |  |
| LSM ≥ 9.6 kPa, n (%) | 57 (31) | 18 (37.5) | | | | 30 (33) | | | 2 (14.3) | 6 (24) | | 3 (50) | | |  |  |  |  |
| LSM ≥ 13 kPa, n (%) | 55 (29.9) | 18 (37.5) | | | | 19 (20.9) | | | 6 (42.9) | 10 (40) | | 2 (33.3) | | |  |  |  |  |

***Table 2.*** Baseline characteristics of patients with ≥significant liver fibrosis according to etiology

BMI, body mass index; T2DM, type 2 diabetes mellitus ; INR, international normalized ratio; CRP, c-reactive protein; ALT, alanine aminotransferase; AST, aspartate aminotransferase; GGT, gamma-glutamyl transpeptidase; ALP, alkaline phosphatase; LDL-c, low-density lipoprotein cholesterol; HDL-c, high-density lipoprotein cholesterol; CAP, controlled attenuation parameter; FIB-4, fibrosis-4 index; LSM, liver stiffness measurements.

***
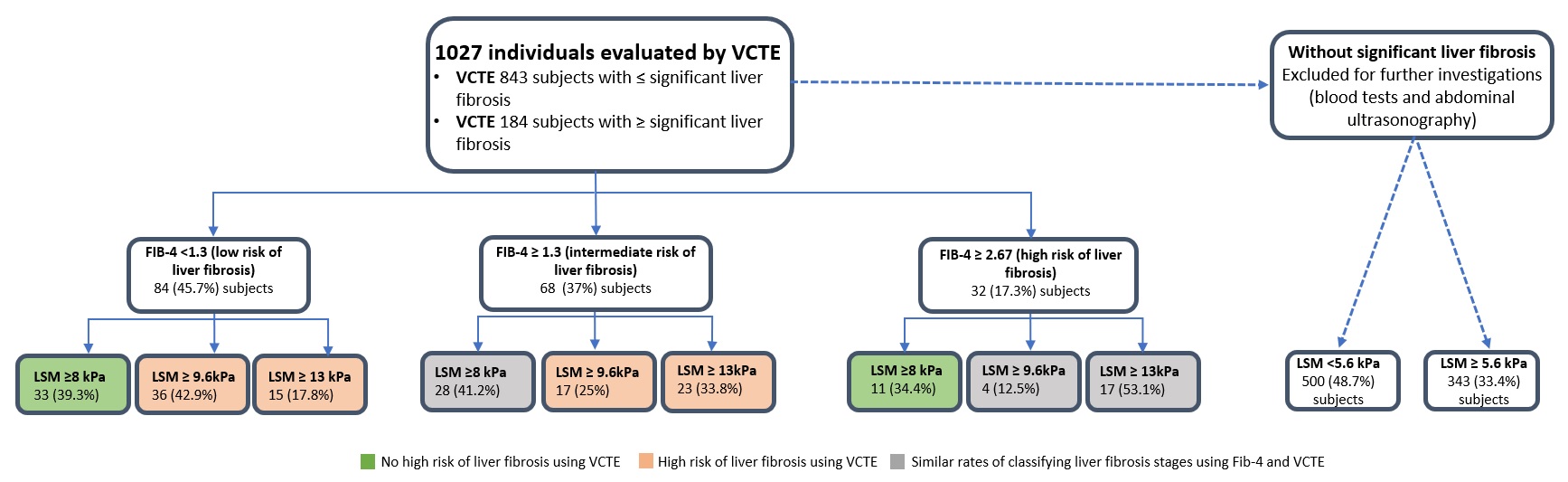
***

VCTE, Vibration-Controlled Transient Elastography; FIB-4, Fibrosis-4 index; LSM, liver stiffness measurements. FIB-4 cut-off values according to age : <65 years, low risk of liver fibrosis (FIB <1.3), intermediate risk of liver fibrosis (FIB ≥1.3), high risk of liver fibrosis (FIB ≥2.67); ≥65 years, low risk of liver fibrosis (FIB <2), intermediate risk of liver fibrosis (FIB ≥2), high risk of liver fibrosis (FIB ≥2.67)

***Figure 2*.** Prevalence of LSM ≥ 8 kPa according to FIB-4 index values
